# Interactions with DCAF1 and DDB1 in the CRL4 E3 ubiquitin ligase are required for Vpr-mediated G_2_ arrest

**DOI:** 10.1186/1743-422X-11-108

**Published:** 2014-06-09

**Authors:** Yoshiyuki Hakata, Masaaki Miyazawa, Nathaniel R Landau

**Affiliations:** 1 Department of Microbiology, New York University School of Medicine, 522 First Avenue, New York, NY 10016, USA; 2 Department of Immunology, Kinki University Faculty of Medicine, 377-2 Ohno-Higashi, Osaka-Sayama, Osaka 589-8511, Japan

**Keywords:** Vpr, DCAF1, DDB1, SIV_agm_ Vpr species-specificity

## Abstract

**Background:**

HIV-1 Vpr-mediated G_2_ cell cycle arrest is dependent on the interaction of Vpr with an E3 ubiquitin ligase that contains damage-specific DNA binding protein 1 (DDB1), Cullin 4A (Cul4A), DDB1 and Cul4-associated factor 1 (DCAF1), and Rbx1. Vpr is thought to associate directly with DCAF1 in the E3 ubiquitin ligase complex although the exact interaction pattern of the proteins in the complex is not completely defined. The Vpr of SIV_agm_ induces G_2_ arrest of cognate African Green Monkey (AGM) cells but not human cells. The molecular mechanism by which SIV_agm_ Vpr exhibits its species-specific function remained unknown.

**Methods:**

Physical interaction of proteins in the E3 ubiquitin ligase complex was assessed by co-immunoprecipitation followed by western blotting. In addition, co-localization of the proteins in cells was investigated by confocal microscopy. The cell cycle was analyzed by propidium iodide staining and flow cytometry. DNA damage response elicited by Vpr was evaluated by detecting phosphorylation of H2AX, a marker for DNA damage response.

**Results:**

We show that RNAi knock-down of DCAF1 prevented the co-immunoprecipitation of DDB1 with HIV-1 Vpr while DDB1 knock-down did not influence the binding of Vpr to DCAF1. HIV-1 Vpr mutants with a L64P or a R90K mutation maintained the ability to associate with DCAF1 but did not appear to be in a complex with DDB1. SIV_agm_ Vpr associated with AGM DCAF1 and DDB1 while, in human cells, it binds to human DCAF1 but hardly binds to human DDB1, resulting in the reduced activation of H2AX.

**Conclusions:**

The identification of Vpr mutants which associate with DCAF1 but only poorly with DDB1 suggests that DCAF1 is necessary but the simple binding of Vpr to DCAF1 is not sufficient for the Vpr association with DDB1-containing E3 ligase complex. Vpr may interact both with DCAF1 and DDB1 in the E3 ligase complex. Alternatively, the interaction of Vpr and DCAF1 may induce a conformational change in DCAF1 or Vpr that promotes the interaction with DDB1. The ability of SIV_agm_ Vpr to associate with DDB1, but not DCAF1, can explain the species-specificity of SIV_agm_ Vpr-mediated G_2_ arrest.

## Background

The Vpr accessory protein is encoded by all lentiviruses but its role in virus replication and pathogenesis is not well understood. Vpr is related by amino acid sequence to the Vpx accessory protein which is encoded by SIV_mac_ and HIV-2. Both Vpr and Vpx are packaged into virions through an interaction with p6 region of HIV Gag [1,2], suggesting a role in the post-entry process of virus replication. Vpr is not required for virus replication in activated CD4^+^ T cells but enhances the ability of the virus to infect macrophages [3,4]. Vpr-deleted SIV_mac_ replicates in Rhesus macaques but tends to revert back to the wild-type, suggesting an important role of this accessory protein in pathogenesis [5,6]. While several functions have been proposed for Vpr such as nuclear import of the preintegration complex, transactivation of viral genes, dysregulation of cellular gene expression, and impairment of mitochondrial functions [7-19], the most widely accepted feature of Vpr function is its ability to induce G_2_ cell cycle arrest [20,21].

Vpr induces G_2_ cell cycle arrest through its association with the E3 ubiquitin ligase CRL4-DCAF1, a complex that consists of the damage-specific DNA binding protein 1 (DDB1), Cullin 4A (Cul4A), the DDB1 and Cul4-associated factor 1 (DCAF1) and Rbx1 [22-28]. DCAF1 is identical to Vpr binding protein (VprBP) that was previously identified [29,30]. The amino- and carboxy-terminal regions of Cul4A are responsible for its interaction with DDB1 and Rbx1, respectively. The adaptor DDB1 links Cul4A to a variety of substrate specificity subunits, DCAFs. Many DCAFs identified so far contain specific WDXR motifs [31-34] and the WDXR motif in DCAF1 has been reported to be necessary for the association both with DDB1 and Vpr [25]. The region of Vpr required for binding to DCAF1 was mapped to the leucine-rich motif within the third alpha-helix domain of Vpr [30]. A Vpr mutant, VprQ65R, which is mutated at 65^th^ amino acid residue in this motif, lost the binding to DCAF1 and the ability to arrest cell cycle at G_2_ phase [25]. In previous reports, the association of Vpr with DCAF1 and DDB1 was examined by co-immunoprecipitation using several Vpr mutants and the results indicated that the binding to DCAF1 fully coincided with the association with DDB1 for all tested mutants [22-24,27]. From these results, Vpr association with DDB1 is thought to be indirectly mediated by simple binding of Vpr to DCAF1.

SIV_mac_ Vpx also contains the carboxy-terminal alpha helix that is conserved in Vpr. Mutation of the conserved glutamine Q76 residue in this motif, which is corresponding to the Q65 residue of Vpr, disrupts the binding of Vpx to DCAF1, suggesting that Vpx uses the conserved helix domain for interaction with DCAF1 in a manner similar to Vpr [25,35]. By interacting with the CRL4-DCAF1 E3 ligase complex, Vpx targets sterile alpha motif domain- and HD domain-containing protein 1 (SAMHD1) for proteasomal degradation to counteract the SAMHD1-mediated lentivirus restriction [36,37]. A recent report resolved the crystal structure of protein complex comprising SIV_sm_ Vpx, the WD40 domain of DCAF1, and the carboxy-terminal region of SAMHD1 [38].

Molecular mechanism by which Vpr induces G_2_ arrest remains unresolved. In particular, a cellular target(s) of the Vpr-CRL4-DCAF1 E3 ligase have not been identified. It has been reported that Vpr activates the DNA damage sensing protein, ataxia telangiectasia mutated and Rad3-related protein (ATR), resulting in the phosphorylation of several proteins including Chk1 and histone 2A variant X (H2AX) [39-43]. A significant portion of Vpr appears to colocalize with the phopshorylated-form of H2AX (γH2AX) in the nucleus [39,44]. The current model for the Vpr-meidated G_2_ arrest is that Vpr binds to the CRL4-DCAF1 complex through DCAF1 to recruit a yet unknown target on the E3 ligase and the recruited target is subsequently ubiquitinated and degraded, resulting in activation of ATR followed by G_2_ cell cycle arrest [22-28]. It was recently reported that untimely activation of the SLX4 complex is involved in the Vpr-mediated G_2_ arrest [45].

SIV_agm_ also encodes the *vpr* gene and SIV_agm_ Vpr induces G_2_ cell cycle arrest in cognate African green monkey (AGM) cells but not in human cells while the Vpr of SIV_mac_ induces G_2_ cell cycle arrest of monkey cells as well as human cells [46,47]. The molecular basis of the species-specificity is still unanswered. In addition to the G_2_ cell cycle arrest, SIV_agm_ Vpr targets SAMHD1 in AGM cells for proteasomal degradation [48].

While it is clear that Vpr or Vpx forms a complex with DCAF1 in the CRL4-DCAF1 E3 ubiquitin ligase, the interaction among these proteins in the ligase complex are not completely defined. Here we show that point mutants of HIV-1 Vpr that maintain their ability to interact with DCAF1 do not associate with the CRL4 E3 ubiquitin ligase, suggesting that simple binding of Vpr to DCAF1 is distinguishable from the association with DDB1 in the E3 ligase. SIV_agm_ Vpr expressed in human cells readily associated with human DCAF1 but only poorly with human DDB1 while it interacted both with AGM DCAF1 and AGM DDB1 in AGM cells. The species-specific dysfunction of SIV_agm_ Vpr in inducing G_2_ arrest in human cells may therefore be caused by its failure to properly associate with DDB1 in the CRL4 E3 ubiquitin ligase.

## Results

### Vpr interaction with the CRL4-DCAF1 complex is dependent upon DCAF1

**Figure 1:**
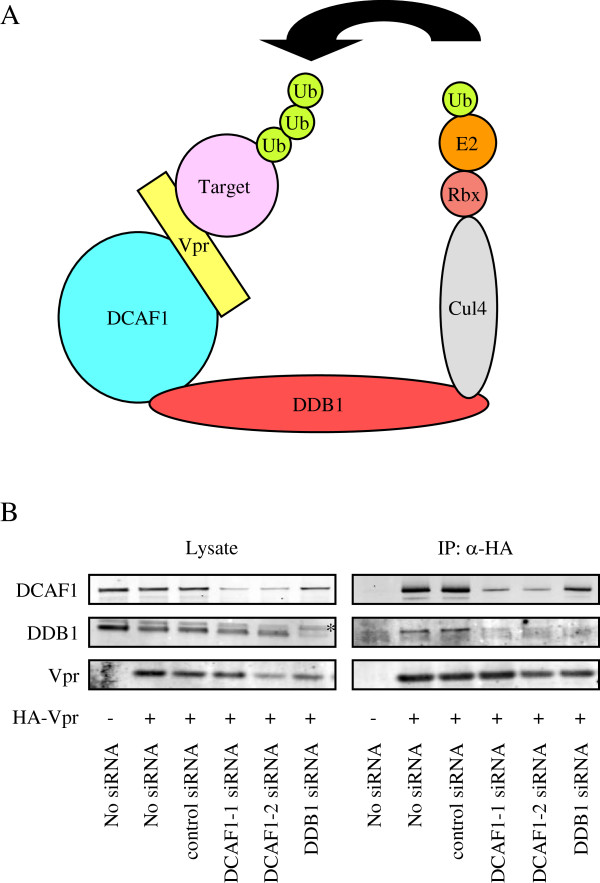
**DCAF1 is required for the association of Vpr with DDB1. (A)** A model of the Vpr-CRL4-DCAF1 E3 ubiquitin ligase complex in which Vpr directly contacts DCAF1. **(B)** HeLa cells were transfected with siRNAs targeting DCAF1 or DDB1. A control siRNA is included and two siRNAs were used against DCAF1. A day later, the cells were transfected again with HA-Vpr expression vector. After another two days, HA-Vpr complexes were immunoprecipitated with anti-HA antibody. Co-immunoprecipitated DCAF1 and DDB1 were detected on an immunoblot. A nonspecific band is indicated with an asterisk.

Vpr is thought to associate with the CRL4-DCAF1 E3 ubiquitin ligase by binding directly to DCAF1 [22-28] as shown in Figure 1A where Vpr binding to DCAF1 is necessary and sufficient for the association. To evaluate this model, we knocked-down DCAF1 or DDB1 and tested whether this affected the ability of Vpr to associate with the CRL4-DCAF1 complex. For this, we transfected HeLa cells with siRNA against DCAF1 or DDB1. A day later, the cells were transfected with pcHA-Vpr, which is an expression vector for HIV-1 Vpr tagged with HA (HA-Vpr). After another two days culture, Vpr was immunoprecipitated with anti-HA MAb and coimmunoprecipitated DCAF1 and DDB1 were detected on an immunoblot. The results showed that the DCAF1 and DDB1 siRNAs knocked-down their respective targets about 80% as compared to a control siRNA which had no effect (Figure 1B). Knock-down of DCAF1 decreased the amount of DDB1 that associated with Vpr. In contrast, knock-down of DDB1 did not affect the amount of DCAF1 associated with Vpr. We also noticed that knock-down of DDB1 caused a small reduction (about 40%) in the steady-state level of DCAF1. This reduction was reproduced with another DDB1 siRNA that targeted a different site on the mRNA (data not shown). The dependence of Vpr on DCAF1 for its association with DDB1 and the lack of dependence of Vpr on DDB1 for the association with DCAF1 suggest that Vpr interacts directly with DCAF1 which mediates the association with DDB1. These results are consistent with the recent model (Figure 1A) showing that DCAF1 is necessary for the association of Vpr with DDB1 in the CRL4 E3 ligase complex.

### HIV-1 Vpr point mutants that bind DCAF1 but poorly bind to DDB1

**Figure 2:**
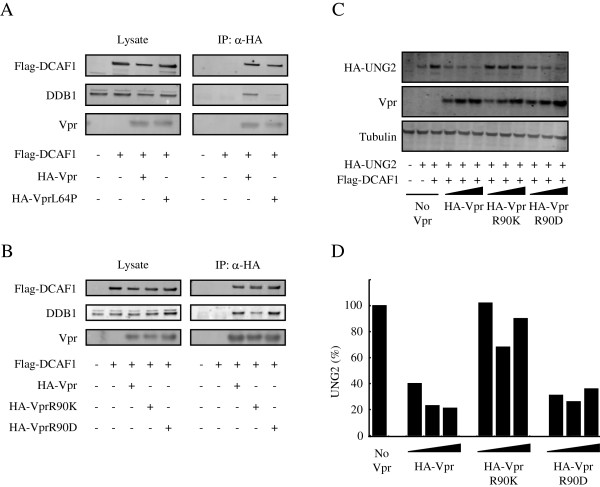
**Vpr-DCAF interaction is not sufficient for Vpr association with DDB1. (A)** 293 T cells were cotransfected with HA-Vpr or HA-VprL64P and Flag-DCAF1 expression vectors. Vpr was immunoprecipitated with anti-HA antibody and the immunoprecipitates were subjected to immunoblot analysis with anti-Flag MAb, anti-DDB1 antibody, and anti-HA antibody. **(B)** HA-Vpr, HA-VprR90K, or HA-VprR90D were expressed with Flag-DCAF1 and were immunoprecipitated with anti-HA antibody. Coimmunoprecipitated DDB1 and Flag-DCAF1 were detected on the immunoblot. **(C)** 293 T cells were transfected with increasing amounts (0.05 μg, 0.1 μg, and 0.2 μg) of HA-Vpr, HA-VprR90K, or HA-VprR90D expression vector together with a constant amount of HA-UNG2 and Flag-DCAF1 expression vectors. Two days later, the cells were lysed and Vpr and UNG2 were detected by immunoblot analysis with anti-HA antibody. The βtubulin was detected as a loading control. **(D)** UNG2 band intensities obtained in **(C)** were quantified and normalized to the UNG2 signal of the third lane from the left in **(C)**. The results are representative data of three independent experiments.

If Vpr binding to DCAF1 which is bound to DDB1 is critical for the Vpr-induced G_2_ cell cycle arrest, Vpr mutants that are defective in G_2_ arrest might fail to bind to DCAF1 and not associate with the E3 ubiquitin ligase complex. Vpr mutants that fall into this category include Q65R and H71R [24,27]. To further test whether mutants that are functionally deficient in arresting cell cycle also fail to bind DCAF1, we evaluated additional mutant Vpr proteins, VprL64P and VprR90K, that have been reported to be defective in inducing G_2_ arrest [26,49]. In addition, we also used a VprR90D mutant in which the charge at amino acid R90 was changed. We transfected 293 T cells with Flag-DCAF1 and HA-Vpr or HA-Vpr mutant expression vectors and tested the possible association of expressed proteins by coimmunoprecipitation. In our experiments we found that the addition of exogenous DCAF1 helped to increase the steady-state levels of the transfected Vprs. We believe that this is because DCAF1 binding is required to stablize the proteins in the cell and that the cell does not contain sufficient endogenous DCAF1 to bind the exogenously experssed Vpr proteins. VprL64P was expressed well and co-immunoprecipitated DCAF1 to a similar extent as wild-type Vpr did (Figure 2A). Unexpectedly, VprL64P co-immunoprecipitated only a small amount of DDB1. VprQ65R is defective for DCAF1 binding [25] and was therefore used as a control. Using this mutant, we confirmed that VprQ65R did not bind to DCAF1 and DDB1 (Additional file 1: Figure S1), confirming the western blot results are reliable. VprR90K and VprR90D were stably expressed and coimmunoprecipitated DCAF1 although VprR90K coimmunoprecipitated less DDB1 compared to wild-type Vpr, a phenotype similar to that of VprL64P (Figure 2B). VprR90D, a nonconservative mutant, retained the ability to coimmunoprecipitate DDB1. To evaluate the ability of VprR90D to arrest the cell cycle at G_2_ phase, we analyzed the cell cycle profile of the transfected cells. Exogenous expression of DCAF1 led to similar expression levels of Vpr and Vpr mutants (Additional file 2: Figure S2B). We found that VprR90D induced G_2_ cell cycle arrest while VprR90K did not (Additional file 2: Figure S2A). These activities are consistent with the association of Vpr mutants with DDB1.

Vpr induces the degradation of uracil N glycosylase (UNG2) [26,50]. UNG2 is a natural substrate for the CRL4-DCAF1 ligase. Although the targeting of UNG2 is not the cause of Vpr-mediated G_2_ cell cycle arrest [49], Vpr is thought to recruit UNG2 to the E3 ligase for degradation [50-52]. To determine whether the mutated Vpr proteins induced UNG2 degradation, we cotransfected 293 T cells with HA-UNG2 and the mutated Vpr expression vectors, and evaluated the steady-state level of UNG2 by immunoblot analysis (Figure 2C and D). VprR90K did not induce UNG2 degradation while VprR90D maintained this function, supporting the idea that VprR90K does not associate well with the CRL4-DCAF1 ligase and is not functional. These findings are not easily compatible with the conventional model (Figure 1A) in which direct binding of Vpr to DCAF1 is sufficient for the interaction of Vpr with DDB1. These results also suggest that Vpr may interact with both DCAF1 and DDB1 in the CRL4-DCAF1 E3 ubiquitin ligase to mediate its function and the two binding sites can be genetically dissociated.

### The species specificity of SIV_agm_ Vpr is caused by inefficient association with DDB1 in human cells

**Figure 3:**
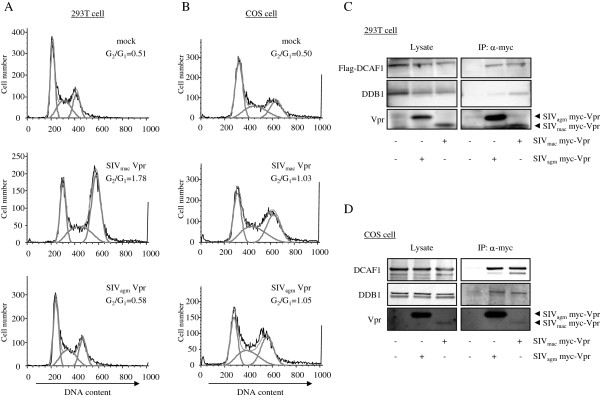
**The species**-**specificity of SIV**_**agm **_**Vpr is correlated with an inability to associate with DDB1 in human cells. (A)** 293 T cells were transfected with SIV_mac_ Vpr or SIV_agm_ Vpr, Flag-DCAF1, and EGFP expression vectors. After two days, the cells were fixed, stained with propidium iodide and analyzed by flow cytometry. The G_2_:G_1_ ratio was calculated after gating for the GFP^+^ cells. The results are representative of three independent experiments. **(B)** COS cells were transfected and the cell cycle profiles were analyzed as in **(A)**. **(C)** 293 T cells were cotransfected with myc-Vpr of SIV_mac_ or SIV_agm_ and Flag-DCAF1 expression vectors. Myc-Vpr was immunoprecipitated with anti-myc MAb and the immunoprecipitated complexes were analyzed on an immunoblot probed with anti-Flag MAb, anti-DDB1 antibody, and anti-myc MAb. **(D)** COS cells were transfected with myc-Vpr of SIV_mac_ or SIV_agm_. Myc-Vpr was immunoprecipitated with anti-myc MAb and coimmunoprecipitated endogenous AGM DCAF1 and DDB1 were detected with anti-DCAF1 and anti-DDB1 antibodies.

SIV_agm_ Vpr has been found to induce G_2_ arrest in African green monkey (AGM) but not in human cells [46,47]. To confirm the species-specificity of SIV_agm_ Vpr, human 293 T and AGM-derived COS cells were transfected with SIV_agm_ Vpr or SIV_mac_ Vpr expression vector and the cell cycle profile was analyzed by flow cytometry. In 293 T cells, SIV_mac_ Vpr induced G_2_ arrest but SIV_agm_ Vpr did not (Figure 3A). On the other hand, both SIV_mac_ and SIV_agm_ Vpr induced G_2_ arrest in COS cells (Figure 3B). These findings confirmed the species-specificity of SIV_agm_ Vpr. To determine whether the specificity is due to species-specific interaction with DCAF1 and DDB1, we tested whether SIV_agm_ Vpr form a complex with DCAF1 and DDB1 in AGM but not human cells. For this, we transfected 293 T and COS cells with SIV_mac_ or SIV_agm_ Myc-Vpr expression vector with (for 293 T cells) or without (for COS cells) Flag-DCAF1 expression vector. We then immunoprecipitated Myc-Vpr and determined the amount of associated DCAF1 and DDB1 by immunoblot analysis. In human cells, SIV_mac_ Vpr coimmunoprecipitated DCAF1 and DDB1 while SIV_agm_ Vpr coimmunoprecipitated DCAF1 but only inefficiently coimmunoprecipitated DDB1 (Figure 3C), a phenotype that was similar to VprL64P. In AGM cells, SIV_agm_ and SIV_mac_ Vpr coimmunoprecipitated DCAF1 and DDB1 (Figure 3D). SIV_mac_ Vpr was expressed at lower levels than SIV_agm_ Vpr but the amount of immunoprecipitated DCAF1 in the samples was similar, suggesting that SIV_mac_ Vpr may bind to DCAF1 more efficiently than SIV_agm_ Vpr. The decreased expression of Myc-tagged SIV_mac_ Vpr was likely not due to protein instability since it pulled-down DDB1 as efficiently as did the highly expressed SIV_agm_ Vpr in COS cells. Thus, in human cells, SIV_agm_ Vpr fails to interact efficiently with complexes that contain DDB1 and does not induce G_2_ arrest. In AGM cells, SIV_agm_ Vpr and SIV_mac_ Vpr associate with DCAF1 and DDB1, and induce G_2_ arrest. These results suggest that the species-specificity of SIV_agm_ Vpr-induced G_2_ arrest is caused by the specificity with which SIV_agm_ Vpr associates with the CRL4 E3 ubiquitin ligase but not due to its specificity with DCAF1.

### SIV_agm_ Vpr does not associate efficiently with DDB1 and induces less of a DNA damage response

**Figure 4:**
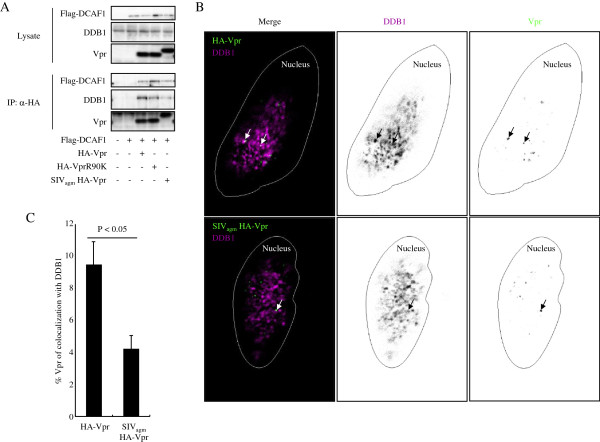
**In human cells, SIV**_**agm **_**Vpr does associates less efficiently with the CRL4 E3 ubiquitin ligase. (A)** 293 T cells were cotransfected with HA-Vpr, HA-VprR90K, or SIV_agm_ HA-Vpr and Flag-DCAF1 expression vectors. Vpr was immunoprecipitated with anti-HA antibody and the immunoprecipitates were analyzed on an immunoblot probed with anti-Flag MAb, anti-DDB1 antibody, and anti-HA antibody. **(B)** HeLa cells were transfected with HA-Vpr or SIV_agm_ HA-Vpr expression vector. The transfected cells were permeabilized, fixed, and then incubated with anti-DDB1 and anti-HA antibodies followed by Alexa Fluor 594-anti-rabbit IgG and Alexa Fluor 488-anti-rat IgG. Representative images from an HA-Vpr or SIV_agm_ HA-Vpr expressing sample are shown. Arrows indicate Vpr foci and DDB1 dots which colocalize. **(C)** The percentage of Vpr foci colocalized with DDB1 among total Vpr foci was calculated. More than 170 Vpr foci were evaluated for each sample and three independent experiments were done. The data are the mean values with standard deviations. P values were calculated by the Student’s t-test with P < 0.05 considered significant.

We next compared the affinity of VprR90K and SIV_agm_ Vpr to DDB1 in human cells. We cotransfected 293 T cells with the respective HA-Vpr expression vector along with Flag-DCAF1 expression vector. Vpr proteins were immunoprecipitated by anti-HA MAb and DCAF1 and DDB1 in the immunoprecipitates were evaluated (Figure 4A). All Vpr proteins including wild type HIV-1 Vpr coimmunoprecipitated DCAF1 at similar efficiency while VprR90K and SIV_agm_ Vpr coimmunoprecipitated less DDB1 as compared to wild type HIV-1 Vpr. Furthermore, the result also showed that the association of DDB1 with SIV_agm_ Vpr is less efficient than that with VprR90K which lost the ability to target UNG2 for proteasomal degradation (Figure 2C).

To further test whether SIV_agm_ Vpr has the reduced association with the CRL4-DCAF1 complex, we cotransfected HeLa cells with HIV-1 or SIV_agm_ HA-Vpr expression vector and assessed the colocalization of Vpr protein with DDB1 by confocal microscopy. Vpr binds to chromatin and forms nuclear foci [39,44]. To visualize Vpr foci, we permeabilized the transfected cells with Triton X100-containing buffer and fixed them. As expected, DDB1, HIV-1 Vpr, and SIV_agm_ Vpr foci were present in the nucleus (Figure 4B). A fraction of the Vpr foci colocalized with DDB1 dot-like signals. We quantified the extent of co-localization by determining the percentage of Vpr foci that were also positive for DDB1 (Figure 4C). The results showed that HIV-1 Vpr colocalized with DDB1 in a higher number of foci than did SIV_agm_ Vpr.

We also anlayzed colocalization of VprR90K with DDB1. As expected, VprR90K colocalized with DDB1 less frequently compared than wild-type Vpr (Additional file 3: Figure S3), confirming its inefficient association with DDB1. The amount of Vpr colocalized with DDB1 was higher than that in Figure 4C, perhaps as a result of stabilization of the protein by DCAF1. Because we detected less association of SIV_agm_ Vpr with DDB1, we tested whether SIV_agm_ Vpr enhances UNG2 degradation. As for VprR90K, SIV_agm_ Vpr failed to induce the degradation of UNG2 (Additional file 4: Figure S4), a finding that is consistent with the idea that SIV_agm_ Vpr does not associate efficiently with DDB1.

**Figure 5:**
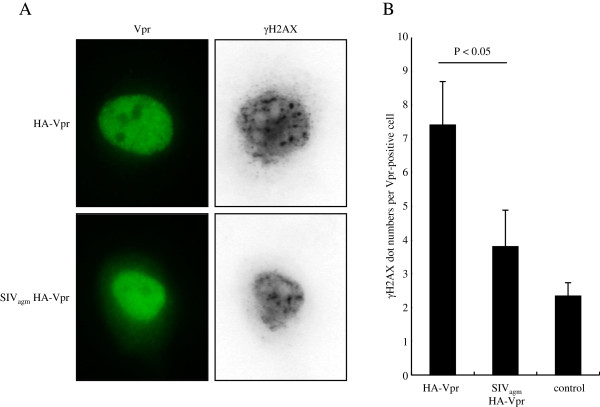
**SIV**_**agm **_**Vpr induces less DNA damage than HIV-1 Vpr. (A)** HeLa cells were transfected with HA-Vpr or SIV_agm_ HA-Vpr expression vector. After two days, the cells were fixed, permeabilized and incubated with anti-γH2AX and anti-HA antibodies. Subsequently, the cells were stained with Alexa Fluor 594-anti-rabbit IgG and Alexa Fluor 488-anti-rat IgG. **(B)** The number of γH2AX dots in Vpr expressing cells was counted. More than 40 Vpr + cells were evaluated in each experiment. Control sample was transfected with pcDNA3.1 instead of Vpr expression vector. The data represent means with standard deviations from three independent experiments. P values were calculated by Student’s t-test with P < 0.05 considered as significant.

Vpr induces a DNA damage response in the cell, which can be detected by phosphorylation of H2AX [42]. The response is initiated through the association of Vpr with the CRL4-DCAF1 ligase. The phosphorylated form of H2AX, γH2AX, forms nuclear γH2AX foci [53]. If SIV_agm_ Vpr associates inefficiently with CRL4, then it should induce fewer γH2AX foci. To test this, we transfected HeLa cells with HIV-1 or SIV_agm_ HA-Vpr expression vector and then counted the number of γH2AX foci in the Vpr-expressing cells (Figure 5A and B). The results showed that SIV_agm_ Vpr induced fewer γH2AX foci than did HIV-1 Vpr. These results were confirmed by immunoblot analysis which showed that Vpr led to an increase in the cellular concentration of γH2AX and that the increase was less pronounced in SIV_agm_ Vpr-expressing cells than HIV-1 Vpr-expressing cells (Additional file 5: Figure S5). Taken together, these data suggest that SIV_agm_ Vpr associates less efficiently with human DDB1 in the CRL4-DCAF1 complex and induces a weaker DNA damage response, resulting in the species-specific deficiency in G_2_ cell cycle arrest.

## Discussion

Our results suggest that the interaction of Vpr with DCAF1 is necessary but not sufficient for Vpr function with respect to G_2_ cell cycle arrest and UNG2 degradation. The configuration of the Vpr-CRL4-DCAF1 E3 ubiquitin ligase complex is more complicated than previously thought since our findings showed that some Vpr point mutants are able to bind DCAF1 but appear not to interact well with the CRL4-DCAF1 complex. In addition, SIV_agm_ Vpr, which has species-specificity in inducing G_2_ arrest, similarly binds to human DCAF1 but does not associate well with the CRL4-DCAF1 complex in human cells.

In support of the previously predicted direct interaction of Vpr with DCAF1, knock-down of DDB1 had no effect on the association of Vpr with DCAF1. Conversely, Vpr did not co-immunoprecipitate DDB1 when DCAF1 was knocked-down with its siRNAs, supporting the idea that DCAF1 is required for the association of Vpr with DDB1. To our knowledge, none of Vpr point mutants that have previously been analyzed were found to associate with DCAF1 but not with DDB1 [22-24,27]. In this study, we found that VprL64P and VprR90K maintained the ability to interact with DCAF1 but only weakly interacted with DDB1. A possible explanation for our findings was that the Vpr point mutants might have failed to localize to the nucleus, as this would have allowed for an interaction with DCAF1 but not with CRL4 which is in the nucleus. However, we found that the mutated Vpr proteins actually localized to the nucleus and their localization was not significantly affected by the overexpression of DCAF1 (data not shown), although overexpression of DCAF1 has been reported to relocalize Vpr to the cytoplasm [54]. Furthermore, the cellular distribution pattern of the Vpr mutants was similar to that of wild-type Vpr in all experimental conditions of this study. Also, the localization of DCAF1 or DDB1 to the nucleus was not affected by wild-type or mutated Vpr (data not shown). Therefore, it is unlikely that the cellular localization pattern of these proteins causes the reduced binding of Vpr mutants to DDB1. Both Vpr point mutants, VprL64P and VprR90K, were defective in inducing G_2_ cell cycle arrest, suggesting that Vpr must associate both with DCAF1 and DDB1 to function. Similar to HIV Vpr point mutants, SIV_agm_ Vpr did not induce G_2_ arrest and had inefficient association with DDB1 despite binding to DCAF1 in human cells. These results further suggest that the binding of Vpr to DCAF1 is not sufficient for the interaction of Vpr with DDB1 in CRL4 E3 ligase complex that mediates the biological function of Vpr.

Our findings suggest that the association of Vpr with DDB1 is accomplished by an additional step(s) beyond the simple binding of Vpr to DCAF1. It is clear that Vpr interacts with DCAF1 but it is possible that it additionally interacts with DDB1, thereby strengthening the complex. The affinity of Vpr for DDB1 alone is not sufficient to be detected in the absence of DCAF1 but may still contribute to the stability of the complex. It is possible that L64 and R90 residues of Vpr are directly involved in formation of the binding interface for this interaction. Alternatively, the interaction with DCAF1 may induce a conformational alteration in Vpr that allows binding to DDB1. Paramyxovirus simian virus 5 and hepatitis B virus encode proteins that bind to DDB1 in CRL4 through an alpha-helical motif [55,56]. Vpr is composed of three alpha-helix domains with flexible elements at both ends that could similarly bind DDB1. L64 and R90 residues may act as a binding interface after the conformational change or may be required for the proper conformational change itself. In this model, DCAF1 binding to Vpr is a prerequisite for the association with DDB1. Another hypothesis is that binding of Vpr to DCAF1 may change the DCAF1 conformation to allow tighter binding to DDB1. Crystallographic analyses may help to better define these interactions.

UNG2 is degraded by CRL4-DCAF1 E3 ubiquitin ligase in the absence of Vpr [52] and Vpr enhances this process. VprR90K was defective for UNG2 degradation most likely because it failed to associate with DDB1. Alternatively, VprR90K could inhibit Vpr-independent UNG2 degradation by sequestering DCAF1 from DDB1-containing CRL4 complexes. In either case, inefficient association with DDB1 could contribute to the failure to degrade UNG2. The inability of VprR90K to degrade UNG2 is inconsistent with a previous report [52] in which VprR90K caused dose-dependent degradation of UNG2. The reason for this difference is not clear, but could be caused by the addition of the exogenous DCAF1 to stabilze the Vpr proteins in our studies.

We have shown that SIV_agm_ Vpr associates weakly with DDB1 in human cells and the inefficient binding correlates with its inability to induce G_2_ arrest. SIV_agm_ replicates well in some types of human cell [57], as Vpr is not necessary for virus replication in T cells. However, the inefficient interaction of SIV_agm_ Vpr with DDB1 could affect virus replication in cell-types such as macrophages where Vpr is thought to play a role.

SIV_mac_ and SIV_agm_ Vpr are only about 30% homologous to HIV-1 Vpr yet interact with the CRL4-DCAF1 complex, highlighting the importance of the E3 ubiquitin ligase for Vpr function. The interaction was species-specific in that SIV_agm_ associated with DCAF1 and DDB1 in AGM cells but only with DCAF1 in human cells. Consistent with this, SIV_agm_ Vpr induced G_2_ arrest in AGM cells but not in human cells. It remains unclear why SIV_agm_ Vpr fails to associate with DDB1 in human cells. Differences in amino acid sequence of AGM and human DDB1 may cause the species-specific interaction. Alternatively, the interaction of SIV_agm_ Vpr with DDB1 may require unidentified cofactors which are differentially expressed in AGM and human cells.

Finally, our findings suggest that analyses of Vpr mutants should determine whether they interact both with DDB1 and DCAF1, as both interactions play a role in function. The related lentivirus accessory protein, Vpx, also associates with the CRL4-DCAF1 ligase and the interaction is required to induce the degradation of the host restriction factor SAMHD1. It will be of interest to determine whether analogous mutations can be identified in Vpx such that DCAF1 binding is retained but interaction with the E3 ubiquitin ligase complex is affected.

## Conclusions

HIV-1 Vpr interacts with DCAF1 and DDB1 to induce G_2_ arrest, a property that is conserved in SIV Vpr. Identification of Vpr point mutants that bind to DCAF1 but only weakly to DDB1 suggests that binding of Vpr to DCAF1 is not sufficient for the formation of Vpr-CRL4-DCAF1 E3 ligase complex and the formation is more complicated processes than previously thought. The species-specificity of SIV_agm_ Vpr in inducing G_2_ arrest is caused by its failure to associate with DDB1 in human CRL4 and subsequent less efficient activation of DNA damage response. The species-specificity of SIV_agm_ Vpr highlights adaptations that the virus has made to replicate in its natural host species.

## Methods

### Expression plasmids

Amino-terminal hemagglutinin (HA)-tagged Vpr expression vectors pcHA-Vpr and pcVprL64P, HA-tagged UNG2 expression vector pcUNG2, myc-tagged SIV Vpr expression vectors pcVpr.agm and pcVpr.mac, and DCAF1/VprBP expression system including pFSZ2-VprBP-FH, pcRev, and pcTat plasmids have been previously described [26,29]. SIV_agm_ Vpr sequence in pcVpr.agm was derived from the SIV_agm.tan_. pEGFP-C1 (Clontech) was used in cell cycle analysis. To construct HA-tagged SIV Vpr expression vector, agm Vpr cDNA was amplified using primers 5′-GGGGATATCATGGCAGAAGGAAGAGATTCCAGG-3′ and 5′-GGGCTCGAGCTATGCAAGTCCTGGAGGAGGCTCTC-3′ from pcVpr.agm template. The amplicon was digested with EcoR-V and Xho-I and cloned into the pcHA-Vpr cleaved with Eco-RV and Xho-I. Expression vectors for VprR90K, VprR90D, and VprQ65R were generated by PCR-based site-directed mutagenesis of pcHA-Vpr. All vectors were confirmed by nucleotide sequence analysis.

### Transfection and immunoblot analysis

Cells were cultured in DMEM supplemented with 10% fetal bovine serum in a CO_2_ incubator at 37°C. For transfections, the cells were seed and transfected the next day using Lipofectamine 2000 (Invitrogen). Cell lysates were prepared using buffer containing 0.5% NP40, 10 mM Tris pH 8.0, 150 mM NaCl, and 2 mM EDTA supplemented with protease inhibitor cocktail III (Calbiochem). Lysates were cleared by centrifugation at 10,000 g for 10 min and the protein concentration was determined by Bradford assay. Lysate (10 μg) was separated by SDS-PAGE and transferred to an Invitrolon PVDF membrane (Invitrogen). The membrane was blocked with 5% non-fat milk in PBS with 0.05% Tween 20 (PBST) and probed with anti-HA MAb 16B12 (1:2,000) (Covance) for 1 h. The membrane was washed with PBST and incubated with ImmunoPure biotin-conjugated goat anti-mouse IgG (1:20,000) (Pierce) for 1 h. As a loading control, the membrane was stained with anti-βtubulin MAb (1:1,000) (Sigma). The filters were treated with Dylight 680-conjugated streptavidin (Pierce) for 30 min and the proteins were detected and quantified on an Odyssey Imager (Li COR). Primary antibodies used for immunoblot analysis following immunoprecipitation were anti-myc MAb 9E10 (1:1,000) (Covance), anti-DCAF1 antibody (1:5,000) (Shanhaigenomics), anti-Flag MAb M2 (1:1,000) (Sigma), anti-HA 3 F10 MAb (1:1000) (Roche), anti-DDB1 MAb (1:1,000) (Zymed), anti-DDB1 antibody C-2 (1:500) (Santa Cruz), anti-HA MAb 16B12, and anti-βtubulin MAb. In some cases horseradish peroxidase (HRP)-conjugated secondary antibodies (Invitrogen) were used and the signals were detected using Luminata Forte Western HRP substrate (Millipore) and LAS1000plus or ImageQuant LAS 4010 systems (Fuji Film and GE healthcare).

### siRNA knock-down

HeLa cells (1 × 10^5^) were transfected with 100 pmol of siRNA (Dharmacon) using Lipofectamine 2000. After 24 hours, the cells were transfected again with 1 μg of pcHA-Vpr. After an additional 2 days, the cells were lysed in IP lysis buffer (1% NP40, 50 mM Tris pH 7.5, 150 mM NaCl, and 2 mM EDTA) supplemented with protease inhibitor cocktail for immunoprecipitation analysis. siRNA target sequences were: DCAF1, 5′-GGAGGGAAUUGUCGAGAAUUU-3′ (DCAF1-1) and 5′-CGGAGUUGGAGGAGGACGAUU-3′ (DCAF1-2); DDB1, 5′-CCUGUUGAUUGCCAAAAAC-3′. The siCONTOROL#3 (Dharmacon) was used as a control siRNA.

### Immunoprecipitation

293 T cells were seeded into 6-well plates (1 × 10^5^/well) and COS cells (1 × 10^6^) were seeded into 10 cm dishes. The cells were transfected at the following day and two days posttransfection, the cells were lysed in 500 μl of IP lysis buffer. The lysates were clarified by centrifugation for 10 min at 10,000 g and precleared with protein-G sepharose. A portion of the cleared lysate was retained for immunoblot analysis and the remainder was incubated with anti-HA MAb 16B12, anti-HA MAb 3 F10, or anti-myc MAb 9E10 antibody bound to protein-G sepharose for 2 h at 4°C. The samples were washed five times with IP lysis buffer and resuspended in SDS-PAGE sample buffer for immunoblot analysis.

### Immunofluorescence

HeLa cells (1 × 10^5^) were grown on a 35 mm glass bottom dish (Matsunami) and transfected with 0.3 μg of pcHA-Vpr expression plasmid. The transfected cells were fixed with 2% paraformaldehyde in PBS for 15 min and permeabilized with 0.1% triton X-100 for 5 min at room temperature. After blocking with 0.5% BSA in PBS for 30 min, the cells were incubated with anti-HA 3 F10 MAb and anti-γH2AX (Abcam) antibodies for 16 hours at 4°C followed by incubation with fluorescent-labeled secondary antibodies. The cells were visualized with a BZ-8000 (KEYENCE) microscope. For colocalization analysis, the transfected cells were permeabilized with 0.05% triton X-100 and fixed in 2% paraformaldehyde. After blocking with 0.5% BSA, the cells were incubated with anti-HA 3 F10 MAb and anti-DDB1 (Abcam) antibodies, and then stained with fluorescent-labeled secondary antibodies. The cells were imaged on a Zeiss LSM510 Meta laser scanning confocal microscope (Zeiss). The secondary antibodies used are Alexa Fluor 488-conjugated goat anti-rat IgG (H + L) and Alexa Fluor 594 goat anti-rabbit IgG (H + L) (Invitrogen).

### Cell cycle analysis

Cells were cotransfected with Vpr expression vectors and pEGFP-C1 vectors. Two days posttransfection, the cells were fixed in cold 75% ethanol, washed with PBS and treated with 0.5 mg/ml RNase A for 30 min. The cells were then stained with 0.05 μg/ml propidium iodide for 30 min and analyzed by flow cytometry in that the GFP^+^ cells were gated to focus on those that were successfully transfected. The data were analyzed using FlowJo 8.5.2 software (Tree star).

## Abbreviations

DDB1: The damage-specific DNA binding protein 1; AGM: African green monkey; ATR: ATM and Rad3-related protein; DCAF1: DDB1-and CUL4-associated factor 1; VprBP: Vpr binding protein; Cul4A: Cullin 4A; HA: Hemagglutinin; SAMHD1: Aterile alpha motif domain- and HD domain-containing protein 1; H2AX: Histone 2A variant X.

## Competing interests

The authors declare that they have no competing interests.

## Authors’ contributions

YH carried out the experiments. YH and NRL conceived of the study, participated in its design, and wrote the manuscript. MM helped to coordinate the design of the study. All authors read and approved the final manuscript.

## Supplementary Material

Additional file 1: Figure S1 Association of VprQ65R mutant with DCAF1 and DDB1. 293T cells were cotransfected with HA-Vpr or HA-VpQ65R and Flag-DCAF1 expression vectors. Vpr was immunoprecipitated with anti-HA antibody and the immunoprecipitates were subjected to immunoblot analysis with anti-Flag MAb, anti-DDB1 antibody, and anti-HA antibody. Click here for file

Additional file 2: Figure S2 Cell cycle analysis of VprR90K and VprR90D mutants. (A) 293T cells were transfected with HIV-1 Vpr or Vpr mutant, Flag-DCAF1, and EGFP expression vectors. After staining with propidium iodide, the cells were analyzed by flow cytometry. The G2:G1 ratio was calculated after gating for the GFP+ cells. The results are representative of three independent experiments. (B) A portion of the cells used in (A) was subjected to an immunoblot analysis to confirm Vpr and Vpr mutants expression. The βtubulin was a loading control. Click here for file

Additional file 3: Figure S3 Colocalization of VprR90K with DDB1. HeLa cells were transfected with HA-Vpr or HA-VprR90K and Flag-DCAF1 expression vectors. The transfected cells were permeabilized, fixed, and then incubated with anti-DDB1 and anti-HA antibodies followed by Alexa Fluor 594-anti-rabbit IgG and Alexa Fluor 488-anti-rat IgG. The percentage of Vpr foci colocalized with DDB1 among total Vpr foci was calculated. More than 380 Vpr foci were evaluated for each sample and three independent experiments were done. The data are the mean values with standard deviations. P values were calculated by the Student’s t-test with P < 0.05 considered significant. Click here for file

Additional file 4: Figure S4 SIVagm HA-Vpr shows a defect in UNG2 degradation. 293T cells were transfected with increasing amounts (0.05 μg, 0.1 μg, and 0.2 μg) of HA-Vpr or SIVagm HA-Vpr expression vector together with a constant amount of HA-UNG2 and Flag-DCAF1 expression vectors. The cells were lysed, and then Vpr and UNG2 were detected by immunoblot analysis with anti-HA antibody. The βtubulin was a loading control. Click here for file

Additional file 5: Figure S5 Phosphorylation of H2AX by Vpr. HeLa cells (1 x 10^5^) were transfected with 0.1 μg of HA-Vpr or SIVagm HA-Vpr expression vector. Twenty-four hours after transfection, the cells were lysed in sample buffer. Vpr and γH2AX in the cell lysate were detected by immunoblot analysis. The βtubulin was a loading control. Click here for file
