# Supplementary figures and images for: Interactions with DCAF1 and DDB1 in the CRL4 E3 ubiquitin ligase are required for Vpr-mediated G2 arrest
Source: Virol J. 2014 Jun 9;11:108. doi: 10.1186/1743-422X-11-108 (PMC4058697; doi:10.1186/1743-422X-11-108)

## Slide 1
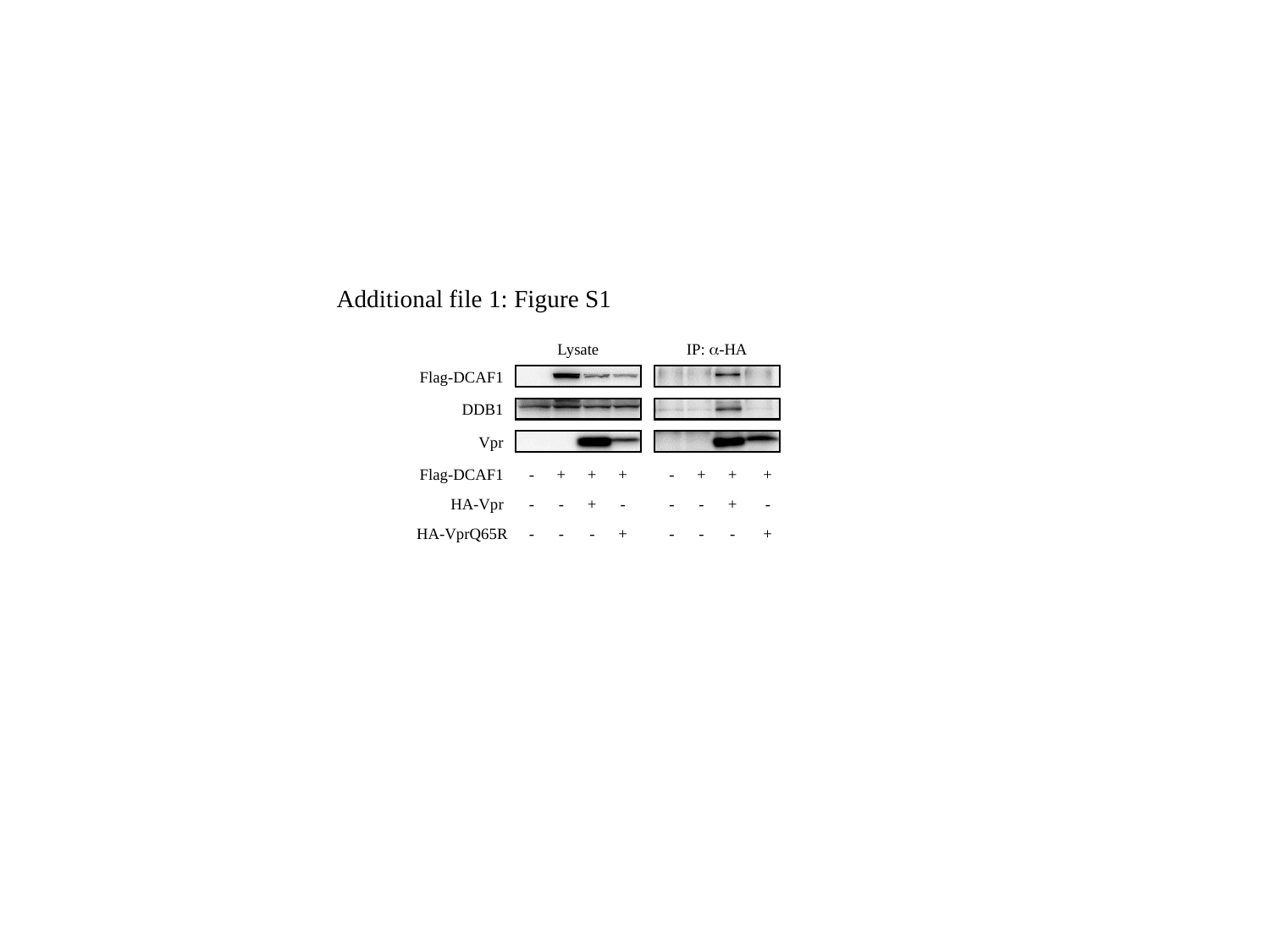

Additional file 1: Figure S1
Lysate
IP: α-HA
Flag-DCAF1
DDB1
Vpr
-
+
+
+
-
+
+
+
Flag-DCAF1
HA-Vpr
-
-
+
-
-
-
+
-
HA-VprQ65R
-
-
-
+
-
-
-
+

Supplement: Additional file 1: Figure S1 — Association of VprQ65R mutant with DCAF1 and DDB1. 293T cells were cotransfected with HA-Vpr or HA-VpQ65R and Flag-DCAF1 expression vectors. Vpr was immunoprecipitated with anti-HA antibody and the immunoprecipitates were subjected to immunoblot analysis with anti-Flag MAb, anti-DDB1 antibody, and anti-HA antibody. [file 1743-422X-11-108-S1.ppt]

## Slide 1
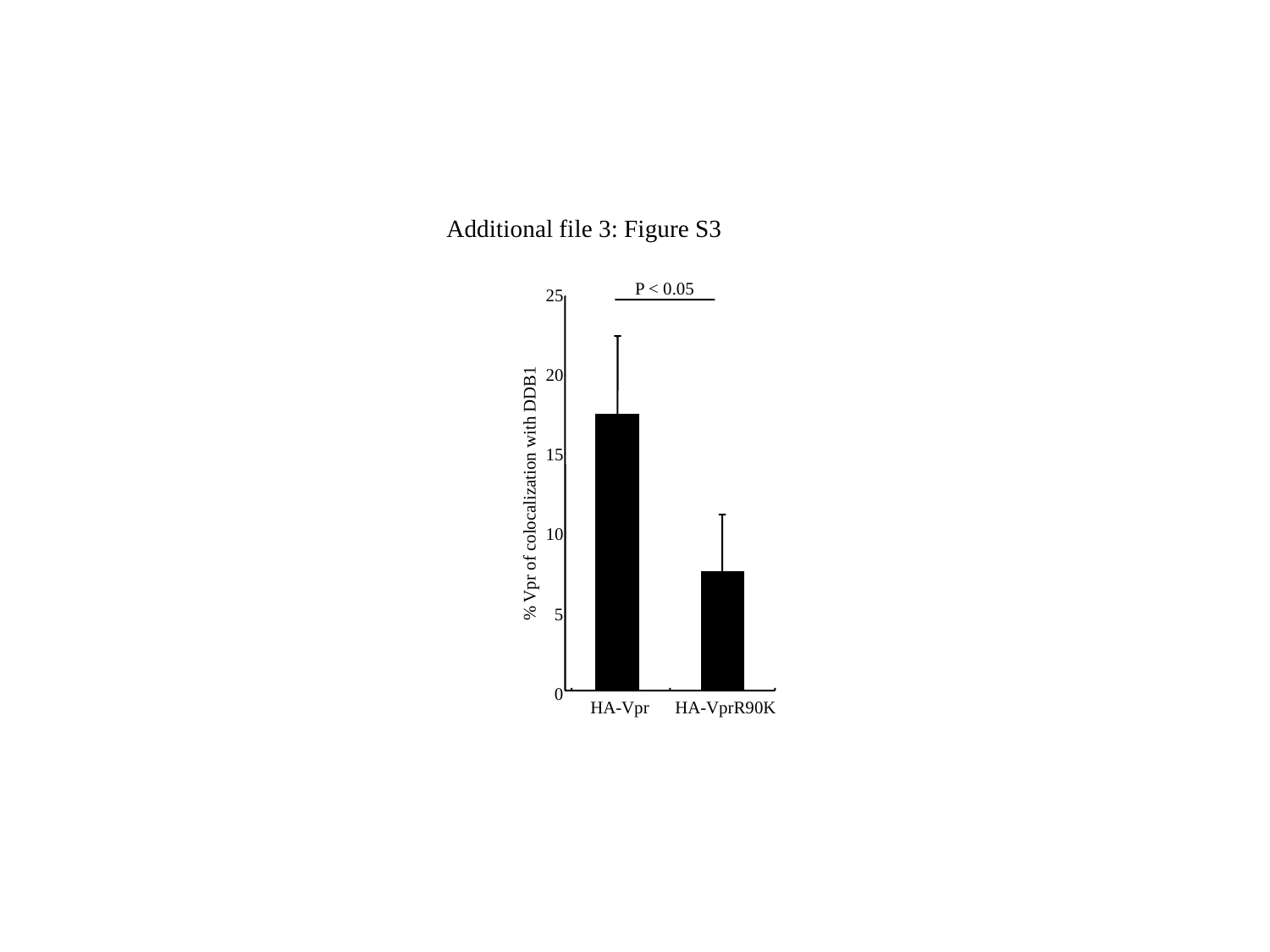

Additional file 3: Figure S3
P < 0.05
25
20
15
% Vpr of colocalization with DDB1
10
5
0
HA-Vpr
HA-VprR90K

Supplement: Additional file 3: Figure S3 — Colocalization of VprR90K with DDB1. HeLa cells were transfected with HA-Vpr or HA-VprR90K and Flag-DCAF1 expression vectors. The transfected cells were permeabilized, fixed, and then incubated with anti-DDB1 and anti-HA antibodies followed by Alexa Fluor 594-anti-rabbit IgG and Alexa Fluor 488-anti-rat IgG. The percentage of Vpr foci colocalized with DDB1 among total Vpr foci was calculated. More than 380 Vpr foci were evaluated for each sample and three independent experiments were done. The data are the mean values with standard deviations. P values were calculated by the Student’s t-test with P < 0.05 considered significant. [file 1743-422X-11-108-S3.ppt]

## Slide 1
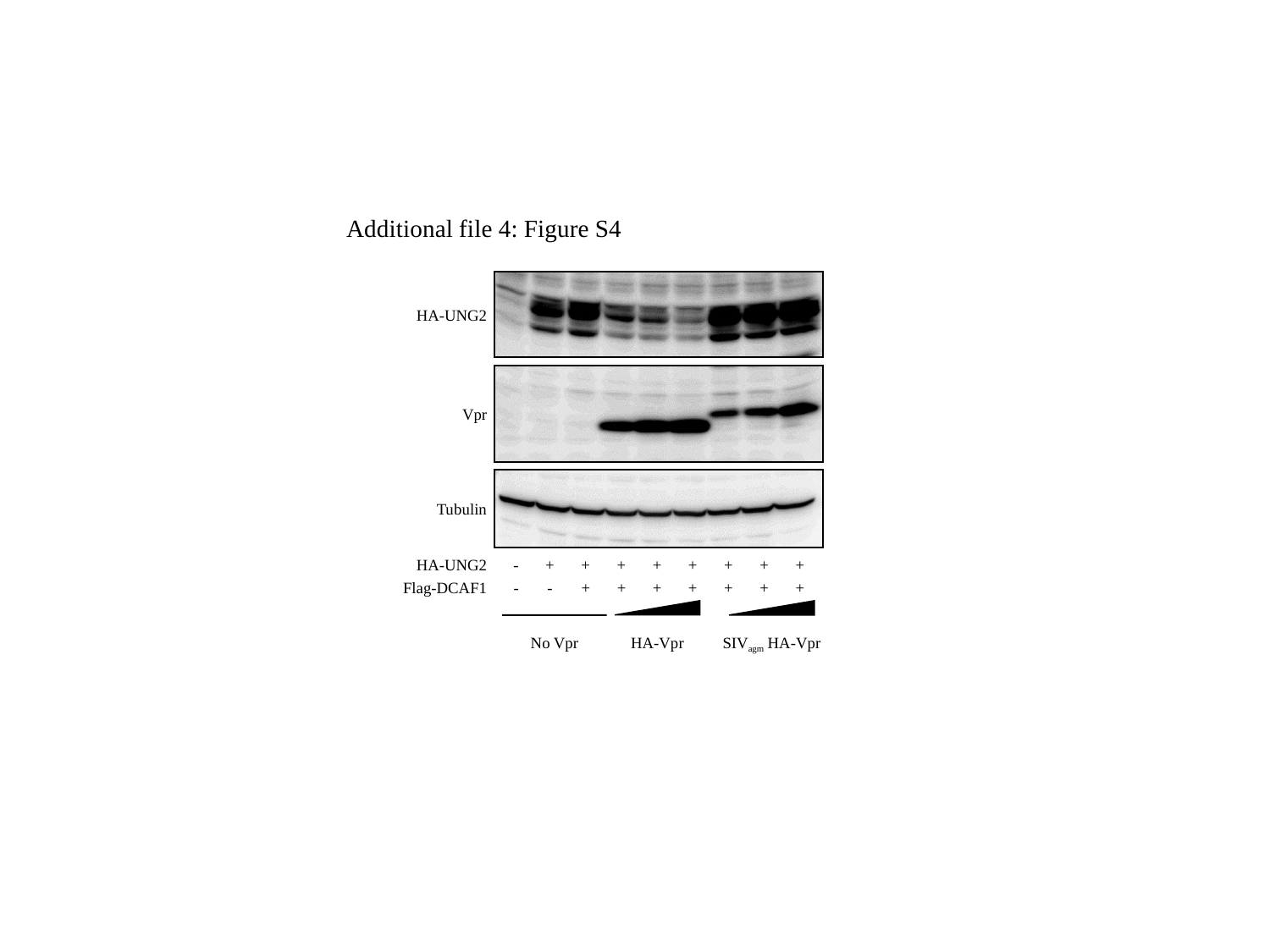

Additional file 4: Figure S4
HA-UNG2
Vpr
Tubulin
HA-UNG2
-
+
+
+
+
+
+
+
+
Flag-DCAF1
-
-
+
+
+
+
+
+
+
HA-Vpr
SIVagm HA-Vpr
No Vpr

Supplement: Additional file 4: Figure S4 — SIVagm HA-Vpr shows a defect in UNG2 degradation. 293T cells were transfected with increasing amounts (0.05 μg, 0.1 μg, and 0.2 μg) of HA-Vpr or SIVagm HA-Vpr expression vector together with a constant amount of HA-UNG2 and Flag-DCAF1 expression vectors. The cells were lysed, and then Vpr and UNG2 were detected by immunoblot analysis with anti-HA antibody. The βtubulin was a loading control. [file 1743-422X-11-108-S4.ppt]

## Slide 1
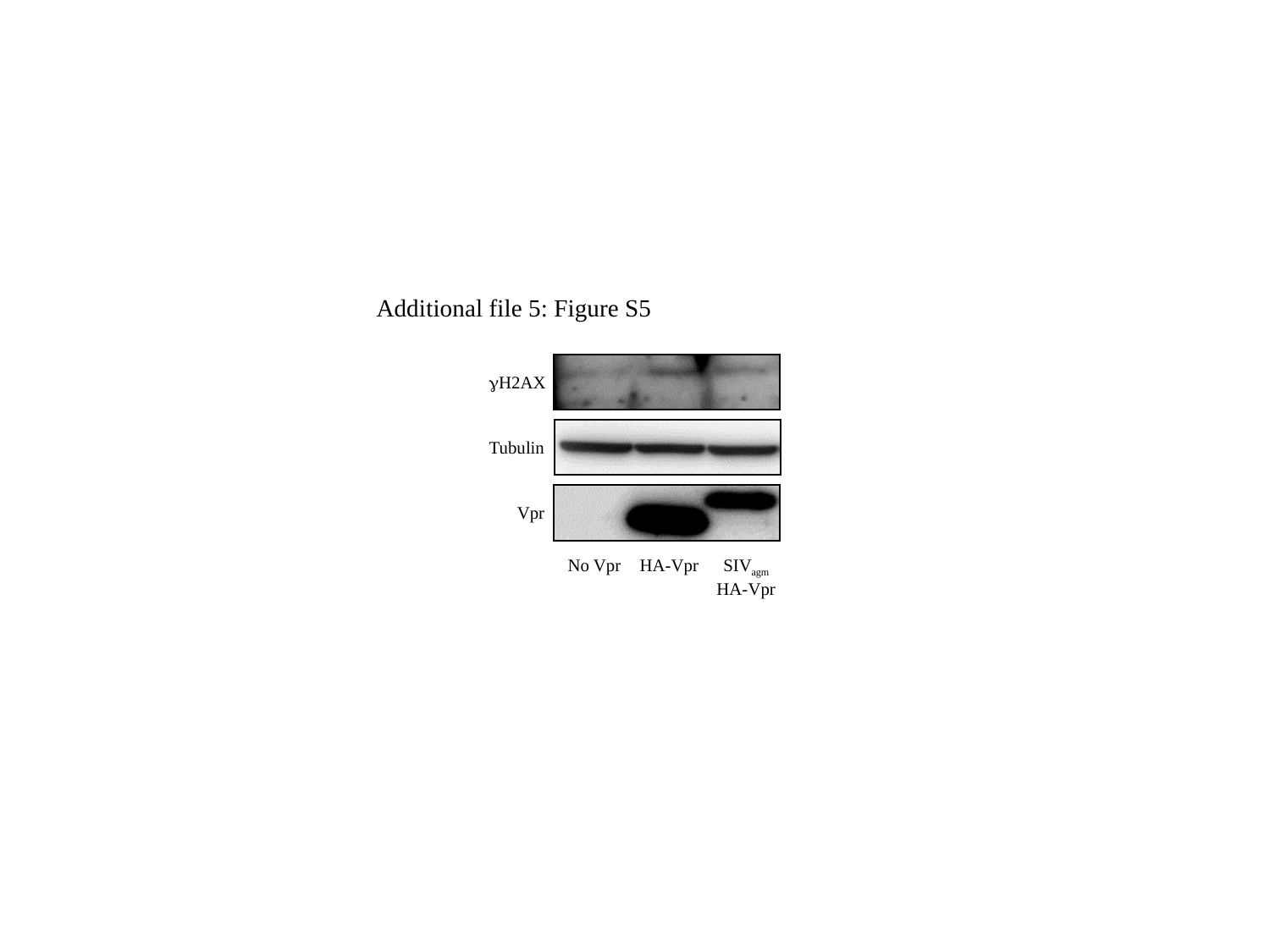

Additional file 5: Figure S5
γH2AX
Tubulin
Vpr
No Vpr
HA-Vpr
SIVagm
HA-Vpr

Supplement: Additional file 5: Figure S5 — Phosphorylation of H2AX by Vpr. HeLa cells (1 x 10^5^) were transfected with 0.1 μg of HA-Vpr or SIVagm HA-Vpr expression vector. Twenty-four hours after transfection, the cells were lysed in sample buffer. Vpr and γH2AX in the cell lysate were detected by immunoblot analysis. The βtubulin was a loading control. [file 1743-422X-11-108-S5.ppt]
