# Supplementary material for: Interactions with DCAF1 and DDB1 in the CRL4 E3 ubiquitin ligase are required for Vpr-mediated G2 arrest
Source: Virol J. 2014 Jun 9;11:108. doi: 10.1186/1743-422X-11-108 (PMC4058697; doi:10.1186/1743-422X-11-108)
Supplement: Additional file 2: Figure S2 — Cell cycle analysis of VprR90K and VprR90D mutants. (A) 293T cells were transfected with HIV-1 Vpr or Vpr mutant, Flag-DCAF1, and EGFP expression vectors. After staining with propidium iodide, the cells were analyzed by flow cytometry. The G2:G1 ratio was calculated after gating for the GFP+ cells. The results are representative of three independent experiments. (B) A portion of the cells used in (A) was subjected to an immunoblot analysis to confirm Vpr and Vpr mutants expression. The βtubulin was a loading control. [file 1743-422X-11-108-S2.ppt]

## Slide 1
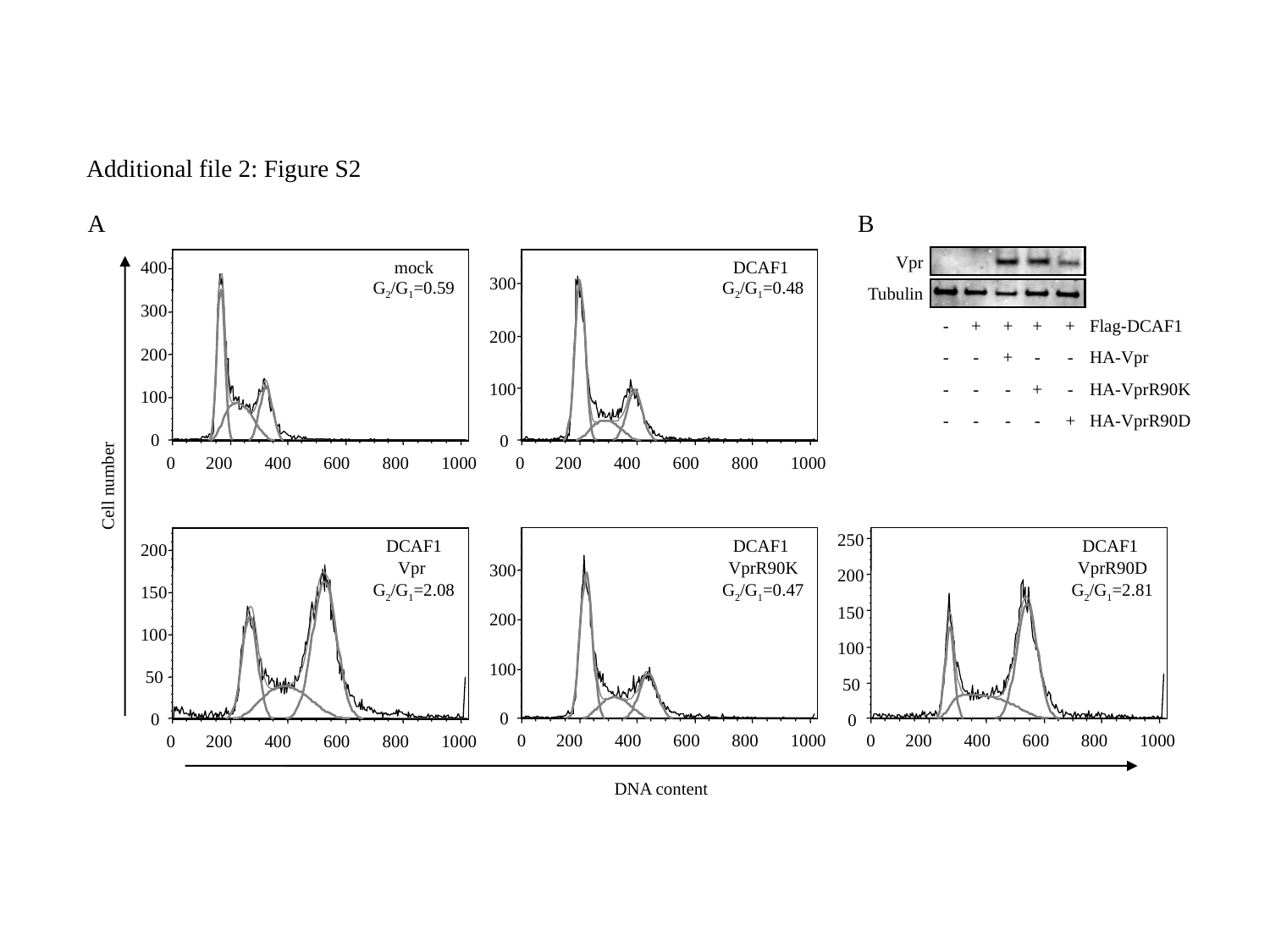

Additional file 2: Figure S2
A
B
Vpr
mock
G2/G1=0.59
DCAF1
G2/G1=0.48
400
300
200
100
0
300
200
100
0
Tubulin
Flag-DCAF1
-
+
+
+
+
-
-
+
-
-
HA-Vpr
-
-
-
+
-
HA-VprR90K
-
-
-
-
+
HA-VprR90D
0
200
400
600
800
1000
0
200
400
600
800
1000
Cell number
DCAF1
VprR90K
G2/G1=0.47
DCAF1
VprR90D
G2/G1=2.81
DCAF1
Vpr
G2/G1=2.08
250
200
150
100
50
0
200
150
100
50
0
300
200
100
0
0
200
400
600
800
1000
0
200
400
600
800
1000
0
200
400
600
800
1000
DNA content
